# Cellulose–Starch Composite Aerogels as Thermal Superinsulating Materials

**DOI:** 10.1021/acsomega.4c05840

**Published:** 2024-12-02

**Authors:** Safoura Ahmadzadeh, Angelina Sagardui, David Huitink, Jingyi Chen, Ali Ubeyitogullari

**Affiliations:** † Department of Food Science, University of Arkansas, Fayetteville, Arkansas 72704, United States; ‡ Department of Chemical Engineering, University of Arkansas, Fayetteville, Arkansas 72701, United States; § Department of Mechanical Engineering, University of Arkansas, Fayetteville, Arkansas 72701, United States; ∥ Department of Chemistry and Biochemistry, University of Arkansas, Fayetteville, Arkansas 72701, United States; ⊥ Department of Biological and Agricultural Engineering, University of Arkansas, Fayetteville, Arkansas 72701, United States

## Abstract

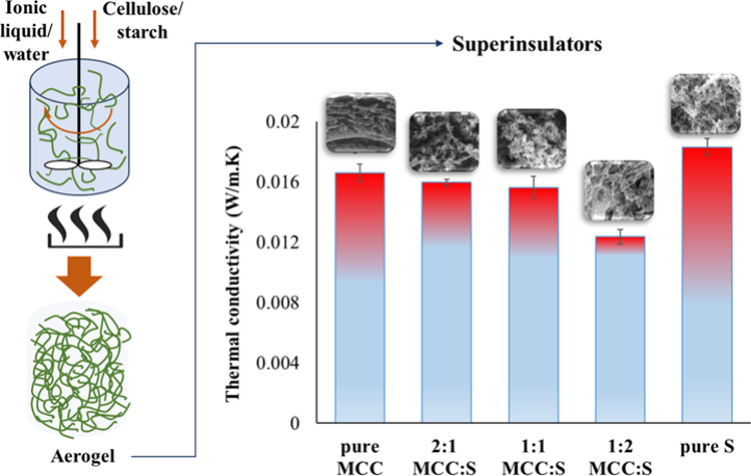

The demand for sustainable packaging materials is rapidly increasing due to growing environmental concerns over the impact of plastic waste. In this study, biodegradable, porous, lightweight, and high-surface-area microcrystalline cellulose–starch (MCC-S) hybrid aerogels were synthesized via supercritical carbon dioxide (SC–CO_2_) drying. The samples were generated using five different MCC-S weight ratios and characterized for their morphology, crystallinity, and structural and thermal properties. When MCC and S were used together, aerogels with superior properties were obtained compared to those made from each component individually. Specifically, the 1:2 MCC-S aerogel exhibited the highest porosity (97%), the lowest density (0.058 g/cm^3^), and the lowest thermal conductivity (0.012 W/(m·K)) along with a high specific surface area (258 m^2^/g). Therefore, MCC-S aerogels are promising insulators for advanced packaging applications, potentially serving as a sustainable alternative to Styrofoam.

## 1 Introduction

Sustainability has grown considerably in popularity among consumers, companies, and governments throughout the past few years. “Green chemistry” is one trend that has emerged from sustainability and focuses on designing products and processes to mitigate harmful effects on human health and the environment.^1^ Two key principles of green chemistry involve reducing toxicity and prioritizing renewability.^2^ Petroleum-based polymers that are frequently employed as insulators in packaging applications (i.e., styrene) are neither biodegradable nor renewable and are recycled at a very low rate. Therefore, the extensive use of plastic foams conflicts with environmentally friendly developments.^3,4^ Furthermore, plastic foams have limited efficiency and do not provide adequate protection against temperature fluctuations.^5^ As a result, the development of biodegradable packaging materials derived from renewable and low-cost resources as an alternative to petroleum-based polymers is required. Recently, there has been growing interest in developing nanoporous structures, such as aerogels, from natural polymers, particularly in applications where biodegradability is essential.

Aerogels are highly porous solid materials with surprising properties, including high porosity and surface area, ultralow density, high refractive index, and low thermal conductivity that make them appealing as superinsulating materials,^6^ filters,^7^ adsorbents, carrier matrix, and medical scaffolds.^8,9^ Nanoporous aerogels can provide more effective protection against temperature fluctuations compared to conventional plastic foams used in packaging due to their low thermal conductivity.

Typically, synthetic polymers (e.g., polyurethane) and inorganic compounds (e.g., silica) have been used to synthesize aerogels.^10^ Recently, with increased attention to sustainable materials and processes, biopolymer-based aerogels have been developed.^9,11^ Biopolymers derived from renewable resources with chemical diversity and distinctive gel properties are of particular interest to aerogel technology. Besides, they present biopolymers’ inherent properties, such as nontoxicity, biocompatibility, and a wide range of biological functions, making them suitable for use in the food and pharmaceutical fields.^12^ Biopolymer-based aerogels are produced using a sol–gel method, which consists of gel preparation, solvent exchange, and SC–CO_2_ drying processes, replacing the solvent with gas while maintaining the structure’s openness. Therefore, the solid phase comprises only 0.2–20% of the total volume.^13,14^ SC–CO_2_ drying is an advantageous method to prepare aerogels in comparison to freeze-drying, as this method eliminates surface tension along the gel wall, thereby better protecting the gel structure and reducing shrinkage.^11,15^ Ahmadzadeh and Ubeyitogullari^9^ reported starch aerogels with significantly lower densities, higher porosities, and larger surface areas via SC–CO_2_ drying compared to freeze-drying.

Cellulose, as the most important inexhaustible biopolymer, has a unique potential for the preparation of multifunctional materials.^16^ Microcrystalline cellulose (MCC) is commercially available in various grades, and it has been successfully used to prepare aerogels with high porosity, also known as aerocellulose.^17^ Sescousse et al.^17^ synthesized cellulose-based aerogels via the sol–gel method followed by drying using a SC–CO_2_ process and compared the properties of aerogels developed from MCC–ionic liquid (IL), MCC–NaOH, and MCC–NMMO systems.^17^ Shi et al. synthesized cellulose cryogels with promising insulation using freeze-drying.^18^ Ahmadzadeh et al. developed nanocomposite foams based on cellulose/surface-modified montmorillonite and evaluated the insulation performance. They reported that the incorporation of nanoparticles improved thermal insulation by reducing pore size.^19^ Several cellulose solvents are known; however, most have limitations or disadvantages, such as being toxic and derivatization of cellulose and not being suitable for all types of cellulose or concentrations. ILs are particularly appealing solvents for cellulose due to their simple dissolution procedure, high thermal stability, and nonvolatility, giving them the designation of “green solvents”.^17^ Furthermore, high molecular weight cellulose can be dissolved in ILs at rather high concentrations.^17^ 1-Butyl-3-methyl immidazolium chloride (BMIMCl) was found to be generally harmless.^20^ As a result, BMIMCl was used as a green, nonderivatizing solvent in this study.

As the main energy reserve in plants, starch is an attractive biopolymer as it is an abundant, biodegradable, sustainable, inexpensive, and food-grade material.^21^ Starches from different sources (potato, pea, tapioca, corn) have been used to prepare aerogels as carriers for controlled release,^9,14,22^ thermal insulators,^6^ packaging materials,^23^ and adsorbents.^24^ High amylose corn starch produces superior aerogels with higher surface area and porosity compared to native corn starch.^9^

MCC, as a filler in starch films, has been shown to improve the thermal, mechanical, and barrier properties of starch-based packaging films by interacting with the starch matrix.^25^ Although several studies have prepared aerogels with only cellulose or starch with promising outcomes,^26^ there are limited studies investigating the mixture of cellulose–starch for generating aerogels with improved properties. MCC–starch composite materials have been used to generate cryogels using freeze-drying;^27^ however, the studies on the formation of aerogels are scarce. Using cellulose and starch together can enhance the properties of aerogels by reducing their density and increasing their porosity.^27^ In addition, starch and cellulose can be found together in several food and agricultural processing byproducts.^28^ Therefore, using these materials to generate high-value, biodegradable aerogels can reduce waste and minimize the additional separation and purification steps.

Since porous structure is a key factor influencing aerogel properties, this study aimed to examine how biopolymer ratio and SC–CO_2_ drying affect the morphological characteristics of composite aerogels and how these changes, in turn, impact their mechanical and thermal properties. The specific objectives of this project were to (i) form nanoporous MCC-S aerogels using SC–CO_2_ drying, (ii) investigate the effect of the MCC-S ratio on aerogel properties, and (iii) characterize the aerogels for their structural, physicochemical, and thermal properties.

## 2 Experimental Section

### 2.1 Materials

MCC powder and corn starch with 55% amylose content were obtained from ThermoFisher Scientific (Ward Hill, MA) and Ingredion Inc. (Bridgewater, NJ), respectively. Pure ethanol was purchased from Koptec (King of Prussia, PA). IL, 1-butyl-3-methylimidazolium (BMIMCl), with 98.0% purity, was purchased from Tokyo Chemical Industry Co. (Portland, OR, USA). Liquid CO_2_ (99.99% purity) was supplied by Airgas, Inc. (Springdale, AR).

### 2.2 Aerogel Formation

**Figure 1:**
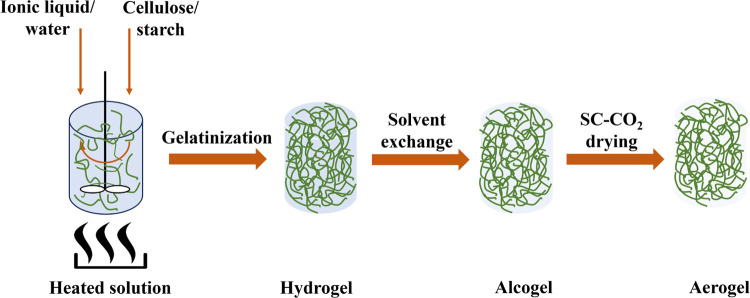
An illustration of key steps in aerogel production.

Figure 1 illustrates the basic processing steps for creating aerogels. First, MCC/starch and IL/water solutions were stirred and heated to induce dissolution and form hydrogels. Then, solvent exchange transformed hydrogels into alcogels. Finally, SC–CO_2_ drying turned the alcogels into aerogels.

#### 2.2.1 Hydrogel Formation

Hydrogels from five ratios of MCC-S (i.e., pure MCC, 2:1 MCC-S, 1:1 MCC-S, 1:2 MCC-S, and pure S) were prepared. Table 1 displays the weight fractions per component for each sample.

**Table 1:** Compositions (%, w/w) of the Hydrogels Generated^a^

	components			
sample	MCC % (w/w)	S % (w/w)	water % (w/w)	IL % (w/w)
pure MCC	2.65			97.35
2:1 MCC-S	1.77	0.88	10.35	87.00
1:1 MCC-S	1.33	1.33	10.35	87.00
1:2 MCC-S	0.88	1.77	10.35	87.00
pure S		2.65	97.35	

a MCC: microcrystalline cellulose, S: starch.

Pure MCC and MCC-S samples were prepared as follows. First, the desired amount of IL/water was weighed and melted at 95 °C. Then, the desired amount of MCC/S was weighed and added to IL, and the mixture was continuously stirred at 95 °C until dissolved. The concentrations of MCC and starch were selected based on preliminary hydrogel formation trials. Upon gel formation, the samples were poured into cylindrical molds and immediately went through solvent exchange.

Pure starch samples were prepared based on the method of Ahmadzadeh and Ubeyitogullari. First, the aqueous suspension of starch was prepared. Then, the dispersion was gelatinized in a Thermomix (Vorwerk, Thousand Oaks, CA) at 95 °C for 30 min at 4260 rpm.^29^ The samples were then poured into cylindrical molds and were immediately placed at 4 °C for 48 h for retrogradation.

#### 2.2.2 Solvent Exchange

Since water and IL do not have high solubility in SC–CO_2_, the solvents were replaced with ethanol, which has a higher solubility in SC–CO_2_, thus turning the hydrogels into alcogels. A five-step solvent exchange was performed at room temperature (23 °C). The samples were soaked in ethanol solutions of 30, 50, 70, and 100% (v/v) for 1 h in each step.^14^ Finally, the samples were soaked in pure ethanol (100%, v/v) for 24 h to ensure a thorough solvent exchange.

#### 2.2.3 SC–CO_2_ Drying

SC–CO_2_ drying was conducted similarly to the procedure of Ahmadzadeh and Ubeyitogullari. An SC–CO_2_ drying system (SFT-120, Supercritical Fluids, Inc., DE) was utilized to remove ethanol from the alcogels, thus, finally generating aerogels. The drying vessel and the restrictor block were set to 50 and 80 °C, respectively. The alcogel samples were placed in the 100 mL drying vessel. The SC–CO_2_ drying was carried out at 10 MPa and 50 °C with a constant CO_2_ flow rate of 1 L/min (measured under ambient conditions) for 4 h. Then, the system was depressurized at the same temperature and CO_2_ flow rate. Afterward, the samples were retrieved and kept in airtight containers at room temperature (23 °C) until additional analyses.^9^

### 2.3 Characterization

#### 2.3.1 Density and Porosity

The weights of the aerogels were determined by using an analytical balance. The dimensions of the samples were measured via a hand caliper to compute the volume. Then, the bulk densities of aerogels were calculated by dividing the weight by volume. Porosity (eq 1) was calculated using the bulk and true densities of MCC and starch, which were measured using a helium pycnometer (Accupyc 1340, Norcross, GA) at 25 °C.^9^
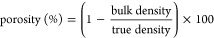
1

#### 2.3.2 Morphology

Scanning electron microscopy (SEM) was conducted using an FEI Nova Nanolab 200 (FEI Company, Hillsboro, OR) dual-beam system equipped with a 30 kV SEM FEG column and a 30 kV FIB column to see the microstructure of the samples.^29^ The samples were prepared for imaging by cutting cross sections and sputter-coating them with gold via an EMITECH SC7620 Sputter Coater (Fall River, MA) to prevent electrical charging. SEM images were taken at an acceleration voltage of 10 kV and a current of 10 mA.

#### 2.3.3 Surface Area, Pore Size, and Pore Volume

Nitrogen adsorption–desorption analysis via an ASAP 2020 (Micromeritics Instrument Corporation, Norcross, GA) was conducted at −196 °C to determine the Brunauer–Emmett–Teller (BET) surface area as well as Barrett–Joyner–Halenda (BJH) pore size and pore volume of the aerogels, according to the method of Ubeyitogullari and Ciftci.^14^ Samples (0.2–0.3 g) were cut into small pieces, placed in the sample tube, and degassed under vacuum at 115 °C for 4 h to prepare for the analysis. The surface area was measured via multipoint BET adsorption characteristics at relative pressures (equilibrium pressure of N2 at the surface of the sample/saturation pressure of N2) of 0.05–0.3. Pore size and volume were determined at relative pressures above 0.35. The overall pore volume was recorded according to the BJH adsorption cumulative volume of pores in the width range of 1.7–300 nm.

#### 2.3.4 Texture Analysis

Mechanical compression tests were carried out via a TA-XT2i Texture Analyzer (Godalming, England, United Kingdom). The experiments were conducted with a 25 mm diameter probe at a rate of 1 mm/s, pressing samples to 90% strain. Compressive strength was determined by dividing the peak force applied by the cross-sectional area of the respective sample. Young’s modulus was obtained from the initial slope on the stress–strain curve.

#### 2.3.5 Crystallinity

X-ray diffraction (XRD) was performed on a PW 3040 X’Pert MRD high-resolution X-ray diffractometer (Philips, Almelo, Netherlands) to analyze the crystallinity of the samples.^29^ Each sample was ground to a fine powder before being placed into the sample holder and then scanned from 5 to 40° (2θ) with a step size of 0.02 at 45 kV and 40 mA.

#### 2.3.6 ATR-FTIR Spectra

Fourier transform infrared spectroscopy (FTIR) was carried out via an IRAffinity-1S FTIR unit (SHIMADZU Corp., Kyoto, Japan) furnished with a Quest attenuated total reflectance (ATR) accessory (Specac Company, Orpington, U.K.).^29^ FTIR spectrum of each sample was obtained in the range of 4000–400 cm^–1^ at a resolution of 4 cm^–1^ with 64 scans.

#### 2.3.7 Thermal Stability

Thermal gravimetric analysis (TGA) was conducted using a Q50 TGA instrument (TA Instruments, New Castle, DE).^14^ Samples (3–5 mg) were put inside aluminum pans and sealed. The samples, equilibrated at room temperature (23 °C) for 10 min, were heated to 600 °C at a rate of 10 °C/min under an N2 gas flow of 20 mL/min. Percent weight loss was recorded and plotted against the temperature. Peak degradation temperature was also determined by finding the first derivative of the TGA plots.

#### 2.3.8 Thermal Conductivity

Thermal conductivity was measured using a handheld 40355 TEMPOS thermal properties analyzer (METER Group Inc., WA). The samples were made in cylindrical shapes with dimensions of 75 mm height and 32 mm diameter. KS3 needle-shaped sensor was embedded in the sample, and the temperature was increased up to 10 °C above the initial temperature. The power level was weak enough not to disturb the effective thermal conductivity of the material.^19^ The experiments were performed at 25 °C and 30% relative humidity.

### 2.4 Statistical Analysis

One-way ANOVA and LSD comparisons of means test (*p* < 0.05) were performed using SPSS Statistics software (version 27, 2020, IBM, Armonk, NY). The means of triplicates, along with their standard deviations, were reported for the measured parameters.

## 3 Results and Discussion

### 3.1 Aerogel Preparation

MCC-based aerogels/cryogels have demonstrated promising properties in previous studies.^30−33^ However, MCC is insoluble in water due to strong intermolecular hydrogen bonding, so IL—which boasts low volatility and low flammability—was used as the primary solvent in this study.^34^ Some deionized water was added to IL in the samples containing starch to improve its gelatinization.^35^

**Figure 2:**
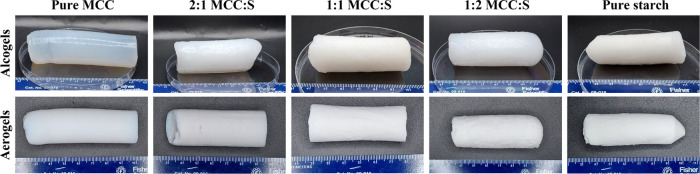
Appearance of alcogels and aerogels made of different MCC/S ratios.

Figure 2 depicts the appearance of the alcogels and aerogels prepared with different MCC-S ratios. Homogeneous alcogels with good firmness for handling were obtained after the solvent exchange. Regardless of the biopolymers ratio, the appearance of samples made from 2:1, 1:1, and 1:2 MCC-S after the solvent exchange and drying was similar. Although the sample made of pure starch appears to be the same as the others, it was weaker for handling during each step. Furthermore, pure MCC alcogel was transparent, which could be attributed to the structure-forming cells that are smaller than the visible light wavelength.^36^

The samples retained their cylindrical shape and shrank slightly during solvent exchange and drying. The gel composition determines the flexibility of fibrils, which influences network resilience during processing.^37^ The findings demonstrated that blending cellulose and starch can improve the properties of aerogels made of only one of them.

### 3.2 Density and Porosity

**Figure 3:**
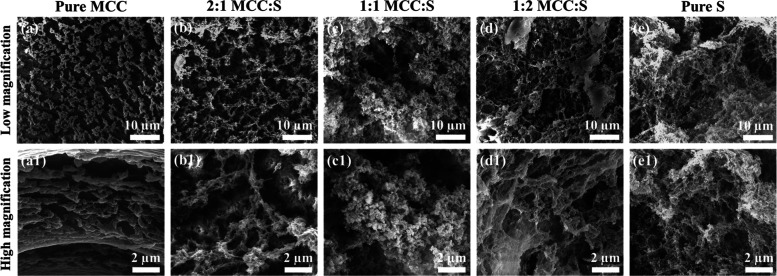
SEM images of MCC-S composite aerogels at low and high magnifications: (a, a1) pure MCC, (b, b1) 2:1 MCC:S, (c, c1) 1:1 MCC:S, (d, d1) 1:2 MCC:S, and (e, e1) pure S. MCC: microcrystalline cellulose; S: starch.

Table 2 presents the density and porosity of aerogels as functions of the MCC-S weight ratio. SC–CO_2_ drying removed ethanol from the matrix while preserving the MCC-S gels’ original three-dimensional network structure, which contributed to their high porosity and low density. The results demonstrated that pure starch aerogel (0.149 g/cm^3^) was the densest, followed by pure MCC aerogel (0.078 g/cm^3^). For the MCC-S composite aerogels, the density decreased with lowering MCC concentration, which can be explained by the effect of starch on the microstructure of aerogels (Figure 3). The presence of starch can prevent the collapse of the MCC skeleton by forming a three-dimensional structure. However, for pure starch, density has increased due to increased agglomeration, resulting in the structure collapsing, as confirmed by the SEM images. Our findings were similar to those of Luo et al., who investigated the properties of starch/MCC cryogels generated using vacuum freeze-drying.^27^ Overall, the 1:2 MCC–starch sample had the lowest density (0.058 g/cm^3^) and highest porosity (∼97%).

**Table 2:** Density, Porosity, Surface Area, Pore Size, and Pore Volume of the MCC-S Aerogels^a^

sample	density (g/cm^3^)	porosity (%)	surface area (m^2^/g)	pore size (nm)	pore volume (cm^3^/g)
pure MCC	0.078 + 0.001^b^	94.62 + 0.62^a,b^	301.97 ± 1.86^a^	11.71 ± 0.70^c^	0.88 ± 0.01^a^
2:1 MCC:S	0.066 + 0.001^c^	95.80 + 0.92^a,b^	251.23 ± 1.48^c^	14.09 ± 1.28^b^	0.81 ± 0.01^c^
1:1 MCC:S	0.062 + 0.001^d^	95.90 + 3.53^a,b^	144.16 ± 0.79^e^	13.78 ± 0.34^b^	0.41 ± 0.01^e^
1:2 MCC:S	0.058 + 0.006^c,d^	96.86 + 0.06^a^	258.65 ± 2.05^b^	12.69 ± 0.99^b,c^	0.84 ± 0.01^b^
pure S	0.149 + 0.002^a^	90.25 + 0.49^b^	147.18 ± 1.07^d^	15.79 ± 0.19^a^	0.61 ± 0.01^d^

a Means that do not share the same letter within the same column are significantly different (*p* < 0.05). MCC: microcrystalline cellulose. S: starch.

The density and porosity of the pure MCC aerogel sample were within the ranges reported by Gavillon and Budtova (2008). However, the density of the pure starch aerogel (0.149 g/cm^3^) was lower compared to the findings of our previous study^9^ (0.48 g/cm^3^), which can be ascribed to the concentration of starch utilized in this study (2.65 vs 12.5%, w/w, respectively).

### 3.3 Morphology

Figure 3 displays SEM images of cross sections of MCC-S aerogels in different weight ratios. All of the samples exhibited a three-dimensional porous network structure. There was no phase separation between MCC and starch, indicating their acceptable compatibility. SEM images at low magnification demonstrate a longitudinal channel structure for pure MCC that was less visible in composite aerogels, particularly in 1:1 and 1:2 MCC:S. In this regard, cellulose might be more capable of providing structural support.

For the MCC–starch composite aerogels, a more porous structure was associated with a decreasing cellulose concentration. The pure MCC, 2:1 MCC:S, and 1:1 MCC:S aerogels had a structure that looked like assembled beads, which could be due to the spinodal decomposition mechanism that occurs due to the coagulation of cellulose by the effect of a nonsolvent.^38^ However, at higher starch content (1:2 MCC:S), a more branched and finer fibrous microstructure was observed (Figure 3). Fewer compact strands observed in the SEM images with decreasing MCC concentration in the MCC–starch composite aerogels contributed to the decrease in their density (Table 2).

### 3.4 Surface Area, Pore Size, and Pore Volume

With lowering MCC concentration, the specific surface area of the hybrid aerogels decreased from ∼302 m^2^/g for pure MCC to 251, 144, and 258 m^2^/g for MCC:S 2:1, MCC:S 1:1, and MCC:S 1:2, respectively; wheras the pore size increased from ∼11 nm for pure MCC to 14, 13, and 12 nm for MCC:S 2:1, MCC:S 1:1, and MCC:S 1:2, respectively. The higher surface area is associated with the nanoscale network structure,^9,39^ which considerably contributes to the pore volume as well. The differences among the surface areas of the aerogels were also observed in the SEM images (Figure 3), where the MCC:S ratio had a significant impact on the microstructure of the aerogels. Cellulose can be coagulated by exposing it to a nonsolvent, and this coagulation was affected by starch, as the strong interaction between starch and cellulose may have prevented cellulose chains from coagulating, resulting in a more branched structure with randomly distributed pores. Furthermore, interactions between cellulose and starch may influence starch retrogradation, resulting in a more porous structure compared to pure starch aerogel. Similar surface areas have been reported for cellulose and starch aerogels.^6,14,40−42^

### 3.5 Texture Analysis

**Figure 4:**
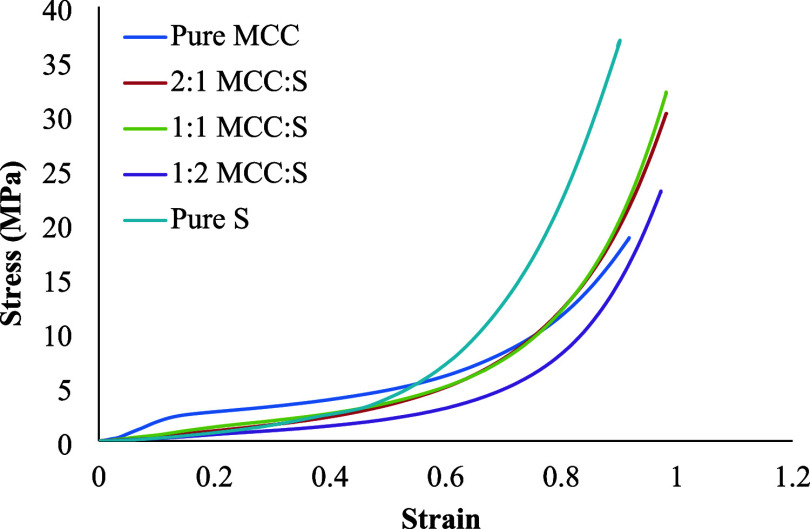
Stress–strain curves for the MCC-S aerogels.

To compare the effect of the MCC-S ratio on the mechanical properties, the aerogels were subjected to a uniaxial compression test. Stress–strain curves of MCC-S aerogels are presented in Figure 4. There was no change in cross-section area during compression for pure MCC, 2:1 MCC-S, and 1:1 MCC-S. However, for 1:2 MCC-S and pure starch, their respective areas were slightly increased. The stress–strain curves revealed three regimes: (a) a linear elastic region used to calculate Young’s modulus; (b) a stress plateau with a lower slope, demonstrating progressive cell wall buckling followed by cell collapsing when reaching the plastic yield; and (c) a densification region, demonstrating wall material compression. The shape of the aerogels was not recovered after the compression test. Table 3 shows Young’s modulus and compressive strength for composite aerogels, which compared well with the literature.^37^ Young’s modulus dropped as MCC content decreased, demonstrating that cellulose increased the resistance of the aerogels to collapse. The pure MCC aerogel had the highest Young’s modulus. Cellulose chains were in close proximity and formed strong intermolecular hydrogen bonds, resulting in stronger structures. The SEM images of pure MCC (Figure 3a,a1) support the observed increased Young’s modulus. The measured values were also comparable to those reported by Sescousse et al., who created aerocellulose by dissolving MCC in an alkaline solvent (i.e., 8% NaOH) or IL (i.e., 1-ethyl-3-methylimidazolium acetate (EMIMAc)). However, it is important to point out that the density of cellulose aerogels has a great impact on Young’s modulus, as described by Sescousse et al.

**Table 3:** Young’s Modulus and Compressive Strength of Aerogels^a^

sample	Young’s modulus (MPa)	compressive yield strength (MPa)
pure MCC	16.00 + 1.20^a^	2.45 ± 0.10^a^
2:1 MCC:S	6.47 + 0.64^b^	1.22 ± 0.12^b^
1:1 MCC:S	5.78 + 0.37^c^	1.21 ± 0.05^b^
1:2 MCC:S	2.32 + 0.96^d^	0.48 ± 0.18^c^
pure S	6.36 + 0.23^b^	2.41 ± 0.07^a^

a Means that do not share the same letter within the same column are significantly different (p < 0.05). MCC: microcrystalline cellulose, S: starch.

### 3.6 Crystallinity

**Figure 5:**
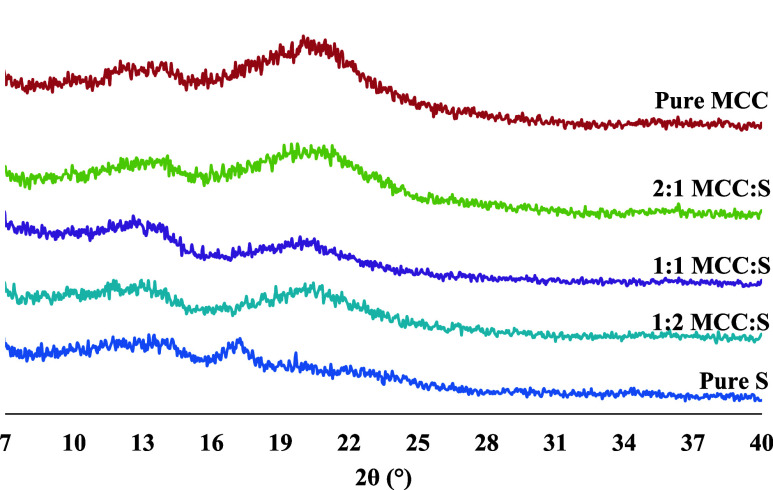
XRD patterns of aerogels prepared with different ratios of MCC and starch.

The crystalline structure of the aerogels generated with different weight ratios of MCC and S was determined by the XRD patterns (Figure 5). The data reveal that after dissolution in BMIMCl and regeneration, the native cellulose type I crystal structure transformed to type II crystal structure associated with two major broad peaks at 12.24 and 20.6°, which is consistent with the results reported by Xu et al.^43^ The diffraction peak at 20.6° was noticeably weaker and broader for 1:2 MCC-S and 1:1 MCC-S aerogels in comparison to pure MCC and 2:1 MCC-S aerogels due to the reduced concentration of cellulose, as well as its reduced crystallinity.

Pure starch exhibited a V-type crystalline structure after gelatinization at high temperatures and retrogradation at 4 °C. A strong diffraction peak was observed at 17.0° for pure starch aerogel, which agrees with the findings of Ahmadzadeh and Ubeyitogullari.^29^ However, this peak does not appear in the XRD patterns of the MCC-S composite aerogels, which could be due to the strong hydrogen-bonding interactions between MCC and starch, resulting in lower crystallinity. In this regard, cellulose linear chains may be more prone to rearrangement, whereas starch chains may be prevented from rearrangement during regeneration due to MCC and starch interactions. In addition, Mateyawa et al.^44^ reported that the gelatinization and dissolution of starch happens competitively when water and an IL are used. They found that at higher water/IL molar ratios gelatinization predominantly occurs, though it is slightly impeded by the strong interaction between water and the IL. They also reported that at water/IL molar ratios of 2.8:1 or 0.1:1, gelatinization occurred at a higher temperature. Considering the ratios of water/IL used in this study, some gelatinization of starch is expected. The SEM and XRD data support this argument. Furthermore, based on our previous research^9^ and literature,^45^ the crystallinities of the MCC and starch reduce after the gel formation step during the aerogel production.

### 3.7 Fourier Transform Infrared Spectroscopy

**Figure 6:**
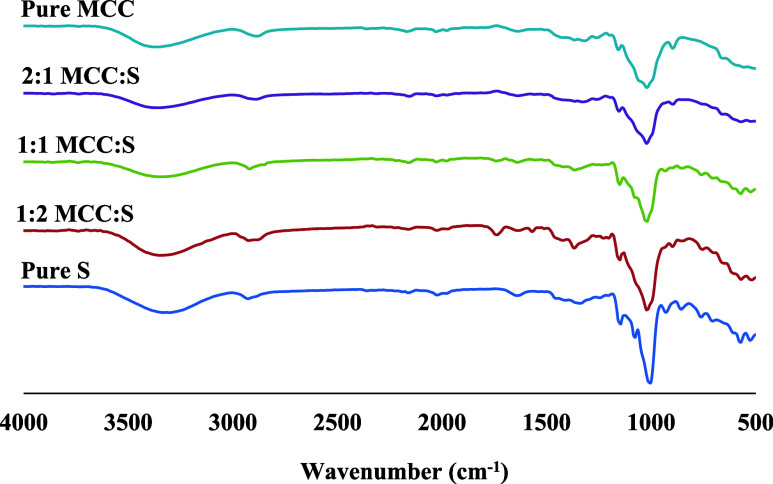
FTIR spectra of aerogels produced via different MCC and starch weight ratios.

The possible chemical interactions in MCC-S aerogels were investigated by using ATR-FTIR spectroscopy (Figure 6). The strong intermolecular and intramolecular H-bonding in the aerogels was reflected by a strong peak in the region of 3600–3000 cm^–1^, which was ascribed to −OH groups stretching mode.^29,43^ Stretching vibration of the −CH_2_ groups was observed in the region of 3000–2800 cm^–1^.^46^ The characteristic FTIR bands ascribed to the C–H stretching vibration appeared at 2885 and 2927 cm^–1^ for pure MCC and pure starch, respectively. These characteristic bands could be seen at 2893, 2920, and 2924 for 2:1, 1:1, and 1:2 MCC:S, respectively. The peak appeared at 894 cm^–1^ was attributed to the vibration of cellulose β-glycosidic linkage.^16,46^ Pure starch aerogel did not show a peak in this region, and the characteristic peak for composite aerogels was shifted to 898 cm^–1^, possibly due to MCC–starch H-bonding interactions.^43^ These strong H-bonding interactions between cellulose and starch may influence the structural supporting function of cellulose (Figure 3).^43^ Furthermore, the absorption bands observed for the composite aerogels closely matched with the characteristic bands observed for MCC and starch, and no new characteristic peaks emerged, which was consistent with previous studies on developing composite films based on cellulose and amylopectin, and on generating gastric-floating drug delivery systems using high amylose starch and MCC.^43,47^

### 3.8 Thermal Stability

**Figure 7:**
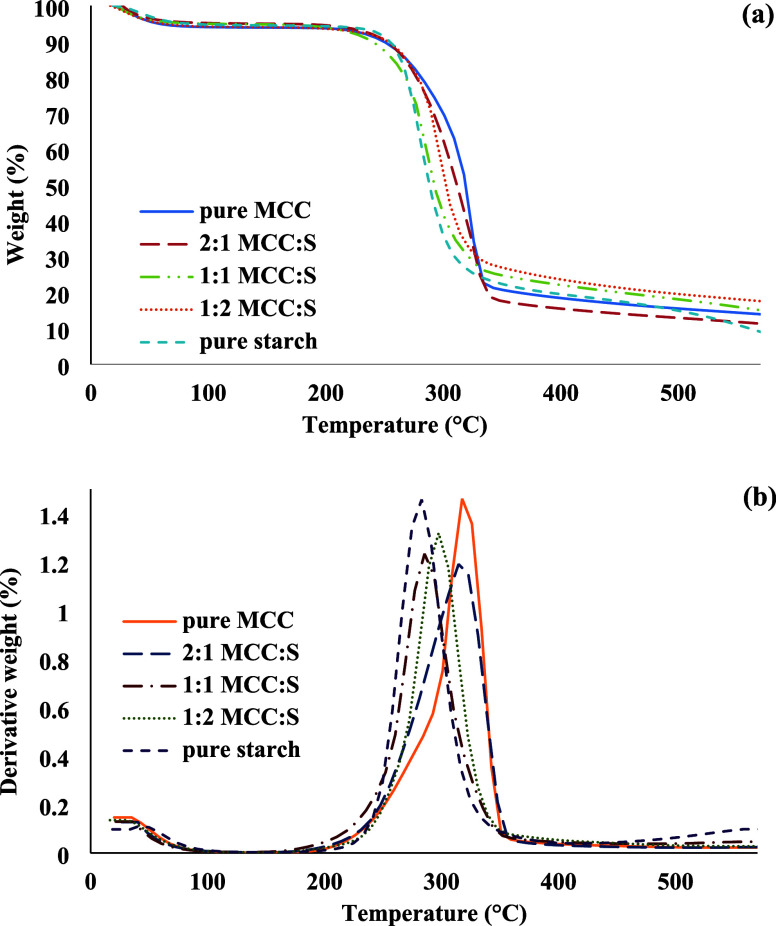
(a) Weight loss and (b) derivative of weight loss curves of aerogels prepared with different ratios of MCC and starch.

The thermal stability of the aerogels was evaluated by using TGA. Figure 7 shows the corresponding profiles of weight loss and derivative thermogravimetry (DTG). Thermal treatment resulted in a two-stage weight loss in all aerogel samples, according to the DTG curves (Figure 7), which were in accordance with prior findings.^48,49^ The first step corresponded to the evaporation–dehydration of water, which occurred within temperatures of 60 to 100 °C. The second stage represented a rapid weight loss in the range of 250–350 °C. Higher peak degradation temperatures indicate better thermal stability. Pure MCC had the highest peak degradation temperature (∼326 °C). These values generally decreased as the MCC concentration decreased. The higher thermal stability of MCC may be attributed to a high cellulose chain orientation and crystallinity. The peak degradation temperatures of the pure MCC and pure starch aerogels were slightly lower than the previously reported values for pure, unprocessed MCC and starch samples,^48,49^ which can be explained with the aerogel formation steps (i.e., gel formation, solvent exchange, and drying).

### 3.9 Thermal Conductivity

**Figure 8:**
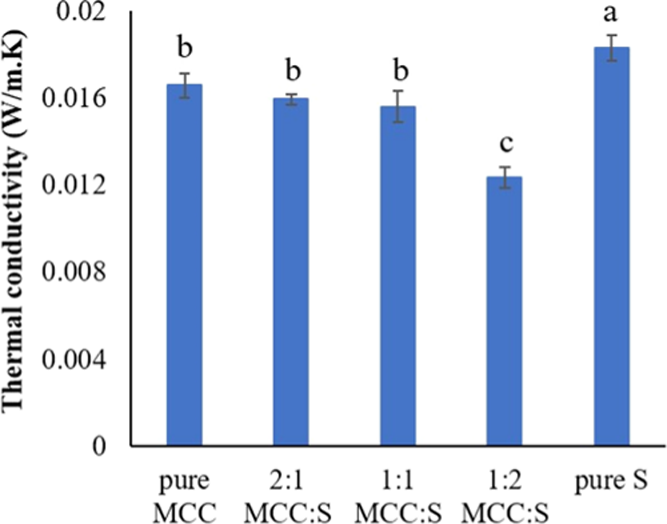
Thermal conductivity values of MCC-S composite aerogels.

Conventional synthetic thermal insulators have low thermal conductivity, which is primarily due to blowing agent properties, morphology, and cell size, with blowing agent type being the most important parameter due to its high-volume fraction.^19,50,51^ The thermal conductivity values for the MCC-S aerogels are shown in Figure 8. The thermal conductivity of 1:2 MCC-S aerogel was much lower (0.012 W/(m·K)) than that of pure MCC and pure starch aerogels. Furthermore, it was found that increasing the starch content in composite aerogels reduced thermal conductivity. The cell structure of the aerogels appears to be the most significant factor influencing thermal conductivity. It has been reported that there is a linear association between thermal conductivity and cell size, with a considerable decrease in thermal conductivity as cell size decreases.^51^ Our findings show that, despite having a larger surface area and smaller pore size, pure MCC aerogel has significantly higher thermal conductivity than the composite MCC:S 1:2 aerogel. This could be due to the microstructure and pore shape. As seen in the SEM images, the pure MCC aerogel has channels in the direction of heat flow, which could increase heat transfer. According to the literature,^52^ the presence of porous channels increases the heat transfer by radiation. The channels can also be seen in MCC:S 2:1, explaining why it has a higher thermal conductivity than MCC:S 1:2. However, the difference in thermal conductivity values of MCC:S 1:1 and MCC:S 1:2 can be attributed to the microstructure of the aerogels and the influence of starch on reducing cell size while creating a more uniform structure and higher surface area. In general, the thermal conductivity values of the aerogels were lower than the thermal conductivity of Styrofoam (0.03 W/(m·K)) reported in the literature.^53^

Thermal conductivity of 1:2 MCC-S was significantly below that of air, confirming the thermal superinsulating properties of the aerogel, which is due to the fine microstructure and low density of the material. The results indicated that a certain ratio of MCC and starch is needed to modify the microstructure of the aerogels and obtain low thermal conductivity. In addition, the values found in the present study were comparable to those reported by Druel et al., who evaluated the structural and thermal properties of starch aerogels.^6^ They reported that pea starch aerogels had reduced thermal conductivity (0.021–0.023 W/(m·K)). Our findings show that cellulose can improve the insulating properties of starch aerogels.

## 4 Conclusions

The effect of the MCC-S ratio (i.e., pure MCC, 2:1 MCC-S, 1:1 MCC-S, 1:2 MCC-S, and pure S) on the aerogel density, microstructure, specific surface area, mechanical and thermal characteristics, and thermal conductivity was investigated. The SEM images illustrated a porous network structure for all aerogels, with the pure MCC aerogel having a fibrous morphology with several compact strands, which became less prevalent with a decreasing MCC concentration. The 1:2 MCC:S aerogel had the lowest density (0.058 g/cm^3^) and thermal conductivity (0.012 W/(m·K)). Conversely, the pure MCC aerogel demonstrated the highest mechanical strength (Young’s modulus of 16.0 MPa) and thermal stability. The FTIR spectra displayed the potential chemical interactions between MCC and starch. Additionally, the XRD patterns suggested that the IL changed the crystalline structure of MCC throughout the aerogel formation process.

The overall impact of this research is that these aerogels can be used as insulators and advanced packaging materials. By sourcing material from biodegradable, renewable components and generating the materials through a green, nontoxic process, the resulting products can potentially be a sustainable alternative to Styrofoam. Furthermore, this process can also add value to food waste and agricultural byproducts, as cellulose and starch are highly abundant in these materials.
